# Effects of inflammatory bowel diseases on sexual function in women

**DOI:** 10.1007/s11845-025-03890-y

**Published:** 2025-01-28

**Authors:** Mubariz Aydamirov, Mustafa Erbayrak, Kadir Karkin, Ediz Vuruskan, Muslum Ahmet Tunckıran

**Affiliations:** 1 Department of Urology, Başkent University Alanya Application and Research Center, Antalya, Türkiye; 2 Department of Gastroenterology, Başkent University Alanya Application and Research Center, Antalya, Türkiye; 3 Department of Urology, Health Sciences University, Adana City Training and Research Hospital, Adana, Türkiye

**Keywords:** Crohn disease, Depression, Sexual dysfunction, Ulcerative colitis

## Abstract

**Background:**

Inflammatory bowel disease (IBD) is a chronic disease that includes Crohn’s disease and ulcerative colitis. Studies found that 40–60% of women diagnosed with IBD have sexual dysfunction (SD).

**Aims:**

To determine SD and associated factors in women with IBD.

**Methods:**

Female patients diagnosed with IBD in the Gastroenterology Department who volunteered and healthy volunteers who were examined by a general practitioner were included in the study as the control group. After appropriate training was provided by the researcher, patients and volunteers were asked to fill out the Clinical and Sociodemographic Questionnaire, Hospital Anxiety Depression Scale and Female Sexual Function Index.

**Results:**

There were 255 patients in the patient group and 240 patients in the control group. The mean ages of the patient and control groups were 40 ± 12 and 38 ± 11 years, respectively. Mean disease duration in IBD patients was 9 ± 5.6 years. The SD rate in the patient group (63.5%) was higher than in the control group (23.8%) (*p* < 0.01). The prevalence of SD was significantly lower in mildly active IBD patients than in moderate and severe IBD patients (*p* < 0.05). Active disease (OR: 3.82), active perianal disease (OR: 2.15), and severe depression (OR: 3.19) were predictive factors for SD in univariate logistic regression analysis. Previous abdominal surgery was found to be predictive for SD in multivariate analysis (OR: 5.13).

**Conclusions:**

The prevalence of SD was high in female IBD patients and its prevalence increased as disease activity increased. History of abdominal surgery in IBD was found to be associated with SD.

## Introduction

Inflammatory bowel disease (IBD) is a chronic disease that includes Crohn’s disease (CD) and ulcerative colitis (UC) [1]. Millions of people in the United States and Europe are affected by this disease [2]. Approximately half of IBD patients are diagnosed before the age of 35 [3]. Improving quality of life is the main goal in the management of the disease. Sexuality is one of the important determinants of quality of life [4]. In a study conducted in Denmark, 90% of participants stated that their sexuality was very important for their health [5]. Studies found that 40–60% of women diagnosed with IBD have sexual dysfunction (SD) [6, 7]. SD also has a major impact on healthcare costs [8].

The prevalence of SD is higher in female IBD patients than in male patients [9]. In female SD, lack of sexual desire, arousal disorder, inability to achieve orgasm, and pain during sexual activity may be observed [10]. Intestinal and extra-intestinal symptoms, vaginal discomfort and rectovaginal fistulas, changes in body image, and poor psychological status may contribute to SD [11, 12]. It is possible that symptoms related to the disease, complications that develop during the disease process and drugs used in treatment may affect sexual function [13]. Depression is common in IBD patients and was reported to be associated with SD [14]. A recent systematic review found that quality of life and fatigue are significant risk factors for SD in female patients with IBD [9]. The effect of disease activity on SD is controversial [15]. While some studies have reported a relationship between disease activity and SD [16], other studies have found that SD is not associated with disease activity [9, 17]. The aims of the study were to: a) compare the prevalence of SD between IBD patient and healthy women, b) compare the prevalence of SD according to disease activity, and c) determine predictive factors for SD in the IBD patient group.

## Materials and methods

This study was conducted prospectively between September 2022 and December 2023. Volunteer female patients diagnosed with IBD in the Gastroenterology Department and healthy volunteers who were examined by a general practitioner were included in the study as the control group.

Female patients who were over 18 years of age, had a stable sexual partner for more than 3 months, were able to understand and complete the questionnaire, and were able to sign the informed consent form were included in the study.

SD before IBD diagnosis, SD due to additional comorbidities (psychiatric/neoplastic/cardiovascular/cerebrovascular/lung/liver/kidney diseases), women who were pregnant/breastfeeding and answered less than 75% of the questionnaires were excluded from the study.

Disease characteristics such as IBD subtype, active perianal disease, abdominal surgery, medication use for IBD, presence of stoma and disease duration were obtained from the patients’ electronic medical files. The aim of the study was explained to all participants. After appropriate training was provided by the researcher, patients and volunteers were asked to fill out the Clinical and Sociodemographic Questionnaire, Hospital Anxiety Depression Scale (HADS), and Female Sexual Function Index (FSFI). All survey forms were coded and kept anonymous. The disease activity score was recorded by the researcher in a different document using the same code as the patient group.

### Assessment of disease activity

The Crohn’s Disease Activity Index (CDAI) [18] and the improved Mayo Score [19] were used. Mild disease was considered CDAI score = 150–220 / Mayo score = 3–5, moderate disease was CDAI score = 220–450 / Mayo score = 6–10, and severe disease was CDAI score > 450 / Mayo score = 11–12.

### Clinical and sociodemographic questionnaire

This included information about patient age, smoking history, weight, height, education level, alcohol consumption, marital status, additional diseases (hypertension, diabetes) and presence of sexual dysfunction (self-described).

### Assessment of anxiety and depression

The Turkish-approved version of the HADS was used to assess the psychological functions of patients [20]. In this form, two scales were assessed: anxiety and depression. A score of ≥ 8 on each scale was defined as anxiety or depression. According to the results of this questionnaire, those who scored ≥ 15 were considered to have severe anxiety/depression [21].

### Assessment of sexual functions

The Turkish validated form of the FSFI was used to assess sexual functions [22]. This form, consisting of 19 questions, evaluated six areas: sexual desire, arousal, lubrication, orgasm, satisfaction and pain in the last 4 weeks (questions 1,2; 3–6; 7–10; 11–13; 14–16; 17–19, respectively). Each question was scored between 0–5 (total score 2–36). Those with a total score of > 26.55 were considered as having SD.

### Ethics approval

This study was approved by Baskent University Institutional Review Board and Ethics Committee (Date: 03/08/2022, Project no: KA22/328). All patients signed written informed consent.

### Statistical analyses

Categorical variables are presented as frequencies and percentages, and continuous variables are presented as means and standard deviations. Continuous variables were evaluated using Student’s T Test. Chi-square test was used to test the relationship between categorical data. Factors affecting SD were examined using logistic regression analysis. Univariate logistic regression analysis was conducted for eligible risks factors for SD in IBD patients. The event of interest was defined as occurrence of SD. Results are presented as odds ratios (OR) with confidence intervals (95% CI). Factors with p-value below 0.10 in univariate logistic regression analysis were included in the full model of multivariate logistic regression analysis. Analyses were performed using IBM SPSS version 26.0 (IBM Corporation, Armonk, NY, USA), and statistical significance was accepted as *p* < 0.05.

## Results

Three hundred patients from each group were invited to participate in the study. In the IBD group, 20 patients refused, 15 patients returned it incomplete, and 10 patients did not return the questionnaire. Out of the healthy controls, 38 individuals did not want to participate in the study, and 22 provided insufficient responses to the questionnaires. In total, 255 patients with IBD completed all questionnaires and 240 patients in the healthy control group participated in the study. Of patients, 147 (57.6%) had CD and 108 (42.4%) had UC. Comparison of clinical and sociodemographic data of all participants is shown in Table 1. The mean ages of the patient and control groups were 40 ± 12 and 38 ± 11 years, respectively. Mean disease duration was 9 ± 5.6 (4–16) years. Among patients, 141 (55.3%) had active disease. Among IBD patients, there were 75 (29.4%) patients with a history of abdominal surgery, 45 (17.6%) patients using corticosteroids, 48 (18.8%) patients using immunosuppressants, 72 (28.2%) patients receiving biological therapy, 18 (7.1%) patients with stoma, and 30 (11.8%) patients with active perianal disease (CD patients with active fissure, fistula, or abscess) at the time of entry to the study. The educational levels of patients and controls were similar (*p* = 0.685). Anxiety, depression, and self-reported SD were higher in the patient group (*p* = 0.004, *p* = 0.001, and *p* = 0.001, respectively) (Table 1).

**Table 1:** Comparison of the demographic and clinical characteristics of patients between the IBD and control groups

	IBD	Controls	*p*
Age, (years)	40 ± 12	38 ± 11	0.723
BMI, (kg/m^2^)	23.9 ± 3.6	25.6 ± 4.2	0.158
Educational level, *n* (%)			0.685
None	9 (3.5)	3 (1.2)	
Primary school	30 (11.8)	24 (10)	
High school	114 (44.7)	96 (40)	
University	102 (40)	117 (48.8)	
Relationship status, *n* (%)			0.349
In a relationship	72 (30.6)	66 (27.5)	
Married	177 (69.4)	174 (72.5)	
Active smoker, *n* (%)	33 (12.9)	27 (11.3)	0.841
Active drinking, *n* (%)	24 (9.4)	21 (8.8)	0.967
Arterial hypertension, *n* (%)	9 (3.5)	6 (2.5)	0.796
Diabetes Mellitus *n*, (%)	3 (1.2)	6 (2.5)	0.283
Anxiety, *n* (%)	63 (24.7)	45 (18.8)	0.374
Depression, *n* (%)	57 (22.4)	9 (3.7)	0.001
Self-reported SD, *n* (%)	36 (14.1)	3 (1.3)	0.001

*IBD* İnflammatory bowel disease; *BMI* Body mass index; *SD* Sexual dysfunction

**Fig. 1:**
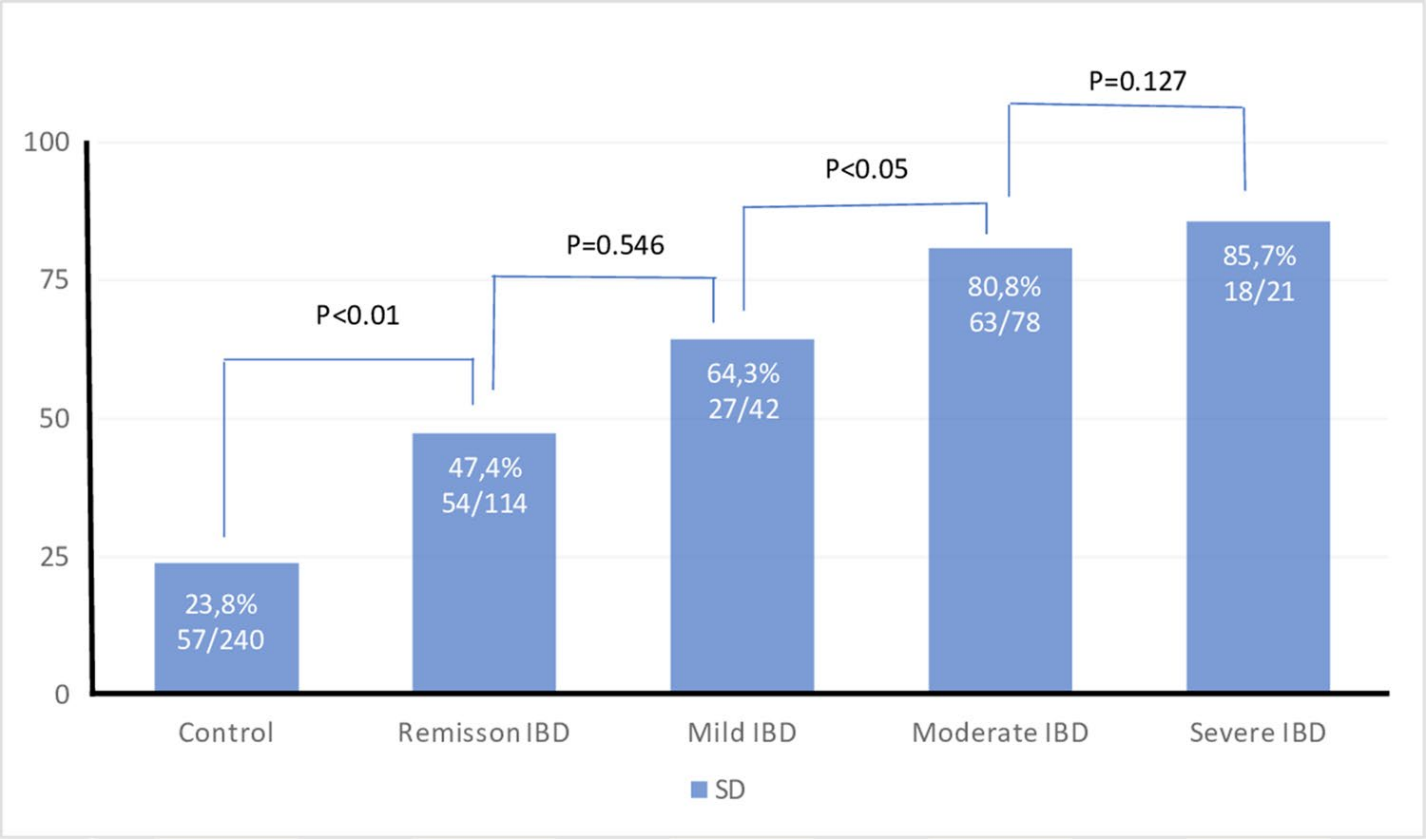
Comparison of sexual dysfunction rates between IBD disease activity levels and control groups according to the FSFI questionnaire. FSFI: female sexual function index, IBD: inflammatory bowel disease, SD: sexual dysfunction

The SD rate in the patient group (63.5%) was higher than in the control group (23.8%) (*p* < 0.01). SD rate was similar between CD and UC patients (65.3% and 61.1%, respectively; *p* = 0.649). The prevalence of SD in active disease was significantly higher than for patients in remission (76.6% and 47.4%, respectively; *p* = 0.023). There was no statistically significant difference in SD between mildly active IBD patients and IBD remission patients (*p* = 0.546). The prevalence of SD was significantly lower in mildly active IBD patients than in moderate and severe IBD patients (*p* < 0.05), but there was no significant difference in SD prevalence between moderately active IBD patients and severe IBD patients (*p* = 0.127) (Fig. 1).

Active disease (OR: 3.82; 95% CI: 1.56–11.39), active perianal disease (OR: 2.15; 95% CI: 1.86–7.94), and severe depression (OR: 3.19; 95% CI: 1.22–11.65) were predictive factors for SD in univariate logistic regression analysis. Previous abdominal surgery was predictive for SD in multivariate analysis (OR: 5.13; 95% CI: 1.72–18.34) (Table 2).

**Table 2:** Factors affecting SD in female IBD patients

	Univariable analysis		Multivariable analysis	
	OR (95% CI)	*P*	OR (95% CI)	*P*
Age	1.01 (0.97–1.05)	0.127		
Body mass index	0.96 (0.85–1.03)	0.743		
Active smoker	0.57 (0.24–2.59)	0.421		
Active drinking	1.37 (0.18–15.34)	0.649		
Disease duration	1.08 (0.99–1.12)	0.185		
Active disease	3.35 (1.56–11.39)	0.021	3.51 (1.32–12.47)	0.105
Active perianal disease	2.15 (1.86–7.94)	0.044	2.37 (1.63–9.25)	0.143
Previous abdominal surgery	4.51 (1.18–12.79)	0.001	5.13 (1.72–18.34)	0.001
Biological therapy	1.04 (1.10–1.87)	0.289		
Corticosteroid therapy	0.86 (0.45–2.76)	0.557		
Immunosuppressant	0.63 (0.22–1.61)	0.265		
Severe anxiety (HADS ≥ 16)	1.53 (0.75–5.92)	0.377		
Severe depression (HADS ≥ 16)	3.19 (1.22–11.65)	0.017	3.38 (1.19–13.92)	0.091

*HADS* Hospital anxiety and depression scale

## Discussion

IBD is a chronic, benign, and relapsing disease with increasing incidence worldwide [23]. It is usually diagnosed in adolescence or early adulthood, when sexuality and fertility are at the forefront [1]. In these patients, scars on the body due to previous surgeries, presence of stoma, excessive steroid use, weight gain, decreased sexual desire due to perianal disease and dyspareunia may be observed [23]. The presence of SD also affects the mental and physical condition of the patients. In this study, it was planned to compare the prevalence of SD between women diagnosed with IBD and the normal population and to investigate the prevalence of SD according to the severity of the disease and the potential risk factors for SD. The results showed that the prevalence of SD was higher in IBD patients compared to the normal population and the frequency of SD increased as the disease activity increased. History of previous abdominal surgery was a predictive factor for SD.

The rates of SD in female IBD patients range from 44.4% to 96.6% [9]. Riviera et al. found SD rates to be 53.6% and 28.3%, respectively, when they compared 192 women diagnosed with IBD and 53 healthy controls [4]. In a study with 130 female patients newly diagnosed with IBD, the prevalence of SD was found to be 97% [3]. Domislovic et al. found the prevalence of SD to be 75% in their study including 80 female patients with a mean disease duration of 11 years [24]. In a recent systematic review including 13 studies, the prevalence of SD in women diagnosed with IBD was 61.4% [9]. In this study, the prevalence of SD in IBD patients was consistent with the literature.

Pires et al. found the active disease rate was 39.7% in 76 female patients [25]. Zhang et al. found the active disease rate was 52.4% [16]. The differences in active disease rates between the literature and this study may be due to differences in the questionnaire forms used for the assessment of active disease and differences in the definition of active disease.

As disease activity increases, fatigue, abdominal pain, and fear of incontinence during intercourse increase, and the frequency of intercourse decreases [17]. In a review, there was no relationship between disease activity and SD, and the disease continued to have a negative effect on sexual functions even in the remission phase [9]. A study including 181 female patients using the Brief Index of Sexual Function in Women found that sexual functions were impaired independent of disease activity [26]. Mules et al. stated that the presence of endoscopically active disease did not affect SD, but active IBD symptoms were associated with SD [15]. Zhang et al. found that the prevalence of SD was significantly higher in severe disease compared to moderate disease in their study of 84 female IBD patients [16]. Active disease causes disturbing symptoms such as fatigue, abdominal pain, and diarrhea [1]. As a result of these symptoms, tension in the pelvic muscles and sexual fear of pain during intercourse develop [6]. Symptoms such as mood changes, feeling unattractive and dissatisfaction with physical appearance in women with active disease are also important risk factors for SD [1]. In this study, those with active disease were divided into three subgroups as mild, moderate and severe. The subgroups were compared among themselves and with the remission and control groups in terms of SD. The prevalence of SD increased as disease activity increased. In this study, there was no significant difference in the prevalence of SD between patients with mild and remission IBD, indicating that mild disease has little effect on sexual function. Although the prevalence of SD was similar among moderate and severe IBD patients, it was significantly higher compared to mild IBD patients.

IBD disease duration is also a risk factor for SD. It was reported that women with disease for more than 3 years have a 2.6-fold decrease in libido [27]. Disease duration was not predictive of SD in this study.

Active perianal disease was found to be a risk factor for SD in IBD patients [16, 28]. Moody et al. reported that dyspareunia, SD and absence of sexual activity due to anal fistula were observed in CD patients [17]. Some studies reported that perianal disease was not a risk factor for SD [4, 15, 25]. In this study, active perianal disease was found to significantly affect SD in univariate logistic regression analysis, but the results were not significant in multivariate analysis.

Ileocecal resection is commonly performed in patients with CD. One study reported increased difficulty with orgasm and dyspareunia risk post-surgery in CD patients [29]. In patients with UC, ileoanal pouch anastomosis (J-pouch) is frequently performed. Since pelvic dissection is done during J-pouch surgery, damage to the autonomic and somatic nerves due to postoperative adhesions may occur [30]. After failed surgeries, increases in stool frequency and fecal incontinence can negatively affect sexual desire, arousal, and satisfaction [31]. In patients unsuitable for a J-pouch, proctocolectomy and ileostomy surgery may be performed [32]. It has been reported that after an ileostomy, 52% of women experience a decrease in sexual attractiveness, and 60% feel less desirable [33]. Mules et al. found that previous abdominal surgery was predictive of SD in their study of 85 female patients [15]. There are also studies showing that abdominal surgery is not associated with SD [9, 16, 25]. These results may be due to differences in surgical methods and differences in the recovery period after surgery. In this study, the presence of abdominal surgery history was a predictive factor for SD in IBD patients.

In a meta-analysis including female IBD patients, the presence of depression was shown to be a predictive factor for SD [9]. Mules et al. found that patients previously diagnosed with depression were not at risk for SD [15]. They stated that this result may be due to improvements in sexual function due to depression treatment [15]. Pires et al. found that depression was predictive for SD in their study including 76 female patients [25]. In this study, although the effect of depression on SD was significant in univariate logistic regression analysis, it was not significant in multivariate analysis.

As clinicians often focus on treating the disease and its systemic consequences, the assessment of SD can be overlooked [34]. During consultations, doctors need to investigate the presence of SD in IBD patients because patients often do not voluntarily report sexual complaints. SD should especially be assessed and managed with a multidisciplinary approach in patients with active disease, depression, and a history of abdominal surgery.

### Limitations of the study

Completion of the study in a single center, disease activity not determined endoscopically, no subgroup analysis for CD and UC patients, no quality of life assessment, no erectile dysfunction assessment among spouses of those in the patient and control group.

## Conclusions

The prevalence of SD was high in female IBD patients and prevalence increased as disease activity increased. History of abdominal surgery in IBD was found to be associated with SD. Sexual functions in female IBD patients should be evaluated and necessary treatments should be provided by clinicians.

## Data Availability

The datasets used and/or analyzed during the current study are available from the corresponding author on reasonable request.
